# Suitability assessment of baseline concentration of MMP3, TIMP3, HE4 and CA125 in the serum of patients with ovarian cancer

**DOI:** 10.1186/s13048-017-0373-9

**Published:** 2018-01-05

**Authors:** Aneta Cymbaluk-Płoska, Anita Chudecka-Głaz, Ewa Pius-Sadowska, Bogusław Machaliński, Janusz Menkiszak, Agnieszka Sompolska-Rzechuła

**Affiliations:** 1 Department of Gynecological Surgery and Gynecological Oncology of Adults and Adolescents, Pomeranian Medical University, Al. Powstańców Wielkopolskich 72, 70-111 Szczecin, Poland; 2 Department of Mathematics Applications in Economy, West Pomeranian University of Technology, al. Piastów 17, 70-310 Szczecin, Poland; 3 General Pathology Department, Pomeranian Medical University, Al. Powstańców Wielkopolskich 72, 70-111 Szczecin, Poland

**Keywords:** Ovarian cancer, Overall survival, PFS, MMP3, TIMP3

## Abstract

**Background:**

MMP and TIMP play an important role in the degradation of extracellular matrix components which are essential for tumor growth, invasion and metastasis. Aim of this research was to assess MMP3 and TIMP3 as prognostic factors among patients with ovarian cancer.

**Results:**

It was found that high levels of output MMP3 correlated with shortened overall survival time of patients by 9.7 months. In addition, it has been shown that high concentrations of output MMP3 were significantly associated with a shorter disease free time in median concentrations implemented *p* = 0.0059. Statistically significant dependence has been shown between an average concentration of TIMP3 protein to the overall survival of patients. The higher output concentration of TIMP3, the longer patients’ survival by 8.9 month. In addition, it was found that high TIMP3 concentrations output were associated with a significantly longer disease free duration at a median concentrations *p* = 0.007.

**Conclusion:**

Preliminary research shows that output levels of MMP3 and TIMP3 proteins correlate with overall survival of patients. In some cases also time free of illness.

## Background

Ovarian cancer is characterized by high mortality rates. There is an ongoing, years-long search for markers that would enable identification of this neoplasm as well as for the prognostic factors that would determine disease course regardless of the management, as well as prediction factors that would indicate possible response to treatment. In 1989 van Houwelingen was the first to introduce a risk-prediction model for ovarian cancer (PI) [1]. Since then, many researchers have tried to create an ovarian cancer prognostic model based on clinical features. Patient characteristics indicating poor prognosis include advanced age, tumor staging, grading, ascites, poorer performance status, residual disease >1 cm [2–4]. In 2015 Zhang added molecular markers: HER2, KRAS, BRCA1, BRAF and EGFR, as independent prognostic factors to the model based on clinical features [5]. For many years, the diagnosis of ovarian cancer was based primarily on the CA125 marker. While introducing additional protein which is HE4, many publications compared their potential. It is currently believed that these two markers complement themselves to the sensitivity and specificity, so that creation of the ROMA algorithm with their contribution has improved the effectiveness of good clinical qualification of patients with ovarian tumor [6, 7]. The role of biomarkers as prognostic and predictive factors has also been considered. In multidimensional analyses, level of CA125 in induction chemotherapy was an independent prognostic factor for optimal cytoreduction [8]. The Ca 125 marker level have also been recognized by some authors as a predictive factor for patients after adjuvant chemotherapy to predict long-term remission [9]. Studies of other authors involving the HE4 marker show that normalization of HE4 levels after the end of chemotherapy was significantly correlated with mortality and higher risk of disease progression [10]. Piovano et al. emphasize the fact that faster recurrence detection is possible with help of HE4 rather than with CA125 [11].

Matrix metalloproteinases (MMP) play an important role in degradation of the extracellular matrix components important for tumor growth, infiltration and metastasis. MMPs are regulated by natural factors known as tissue inhibitors of metalloproteinase (TIMPs).

In our earlier publication, we assessed the possibility of using serum concentration of MMP3 as a diagnostic test for the differentiation of ovarian tumors. The results were satisfactory, however, compared to the currently used markers - CA125, HE4 and ROMA algorithm, it was poorer [12].

It was demonstrated that MMPs are particularly important for the processes of tumor invasion and progression, and elevated preoperative serum MMP2, MMP9, and TIMP1 concentrations constitute a strong prognostic factor of poor prognosis among patients with colon cancer [13]. Literature reports on the role of metalloproteinases and their inhibitors as prognostic and predictive factors in ovarian cancer are lacking. Aim of this research was:

1. To assess MMP3 and TIMP3 as prognostic factors among patients with ovarian cancer.
2. To evaluate serum concentration usefulness of MMP3 and TIMP3 in ovarian cancer diagnostics.

## Methods

A group of 104 female subjects hospitalized at the Clinic of Gynecological Surgery for Adults and Adolescents in years 2011–2014 was included in the study. All patients gave their informed consent for participation in the study. Each patient had been subjected to collection of a 5-mL blood sample before the laparoscopic procedure. It was divided into two test tubes. Determination of MMP3 and TIMP3 was performed at the Laboratory of the Department of General Pathology. Serum concentration of CA125 and HE4 were performed at the Central Laboratory in the Hospital.

Transvaginal ultrasound examination was performed in all patients. In 44 cases of suspected ovarian cancer, CT scans were also performed.

Final distribution into 3 groups were made after receiving a histopathological analysis:

- patient with papillary serous ovarian cancer - 44,
- patient with endometrial ovarian cysts - 30,
- patient with simple cysts - 30,

The group of ovarian cancer patients was stratified according to:

- staging FIGO I, II - 9; FIGO III, IV - 35,
- ovarian cancer grading; low grade - 13, high grade - 31
- with ascites - 28, without ascites - 16,
- primary surgery - 33, neoadjuvant chemotherapy - 11,
- optimal debulking - 26, suboptimal debulking - 7.

Patients were qualified for primary surgery or neoadjuvant chemotherapy during laparoscopic procedure. Following findings were taken into account when qualifying patients into individual groups: omental cake, peritoneal carcinomatosis mesenteric retraction, bowel infiltration, stomach infiltration, and superficial liver metastasis. Following the surgical treatment, patients were divided into patients having undergone radical surgery without residual lesions and patients with residual disease.

MMP3 concentrations were quantified in serum by multiplex fluorescent bead-based immunoassays (Luminex Corporation, Austin, TX, USA) using commercial Human MMP Magnetic Bead Panel 1 (Merck Millipore, Billerica, MA, USA). TIMP3 concentrations were quantified in serum by multiplex fluorescent bead-based immunoassays (Luminex Corporation, Austin, TX, USA) using commercial Human TIMP3 Magnetic Bead Panel 2 (Merck Millipore, Billerica, MA, USA).

CA125 was determined with the Architect i2000 assay from Abbott Diagnostics. The normal range was <35 U/mL. Serum HE4 concentrations were measured using the Elecsys ECLIA assay from Roche running on the cobas e 601 analyzer. The normal range was below 70 pmol/L for premenopausal women and below 140 pmol/L for postmenopausal women.

Statistical analysis was carried out using the Statistica 1.0 software. Results of MMP3, TIMP3, CA125, HE4 determination in individual patient groups and subgroups were presented as medians and ranges. Inter-group comparisons were performed using nonparametric Mann-Whitney U-test. Kaplan-Meier survival analysis and log rank test were used to assess the impact of MMP3, TIMP3, CA125, HE4 on overall survival and time to disease progression. To this date, the following cut-off values were determined for baseline CA125, HE4, MMP3, TIMP3 levels: median, 75th percentile, and 95th percentile. Uni- and multivariate analysis was performed using the Cox regression model. Parameters included in multivariate Cox analysis included age, FIGO stage, tumor grade, residual disease status, as well as median, 75th percentile, and 95th percentile of MMP3, TIMP3, CA125, HE4 levels. The age was assessed as a continuous variable, FIGO stage was stratified as I/II vs. III/IV; grading was stratified as G1 vs. G2 vs. G3, while the residual disease was stratified according to optimal vs. suboptimal surgery. The level of significance was established at *p* < 0.05.

## Results

### Comparative analysis of the groups studied

Mean age of patients and selected serum protein concentrations are presented in Table 1.

**Table 1:** Concentrations of TIMP3, MMP3, HE4 and CA125 in blood serum, depending on the histopathologic diagnosis of patients

variable	ovarian cancer			ovarian simple cysts			*p*
	n	mean range	median 95% CI	n	mean range	median 95% CI	
age	44	61.4 (49–78)	62.2 (52–76.4)	30	58 (42–66)	58.6 (44–62.5)	NS
TIMP3 [pg/ml]	44	138 (67–198)	143 (151–188)	30	285 (148–368)	291 (298–355)	*p* = 0.001
MMP3 [pg/ml]	44	20836 (16211–28994)	21022 (16750–26877)	30	14657 (10987–21003)	15221 (11234–15001)	*p* = 0.0002
HE4 [pmol/l]	44	423 (238–698)	451.6 (250.2–644)	30	54.6 (41–79.6)	57 (44–75.2)	*p* = 0.0004
CA125 [U/ml]	44	728 (432–1254)	790.3 (478.1–1187)	30	19.3 (11.3–37.1)	25 (14.3–35.2)	*p* = 0.0001
variable	ovarian cancer			endometrial ovarian cysts			*p*
	n	mean range	median 95% CI	n	mean range	median 95% CI	
age	44	61.4 (49–78)	62.2 (52–76.4)	30	48.9 (40.2–64.3)	48 (46–64)	NS
TIMP3 [pg/ml]	44	138 (67–198)	143 (151–188)	30	223 (143–276)	241 (256–270)	*p* = 0.003
MMP3 [pg/ml]	44	20836 (16211–28994)	21022 (16750–26877)	30	14111 (10198–19872)	15231 (11263–18862)	*p* = 0.004
HE4 [pmol/l]	44	423 (238–698)	451.6 (250.2–644)	30	61.3 (34–87)	64 (37.6–82.4)	*p* = 0.002
CA125 [U/ml]	44	728 (432–1254)	790.3 (478.1–1187)	30	45.2 (23–98)	47 (24.5–86.2)	*p* = 0.001

Median TIMP3 levels revealed significantly lower concentrations in a group of patients with ovarian cancer compared to patients with endometrial cysts, *p* = 0.001.

Mean concentration of MMP3 (20836 pg/ml) marker as well as CA125 and HE4 (798 U/ml / 423 pmol/L) in the group of ovarian cancer patients was significantly higher compared to mean concentrations of those markers among patients with endometrial cysts.

Comparison of mean TIMP3 levels demonstrated significantly lower concentrations of the marker in the group of patients with ovarian cancer compared to patients with benign cysts (138 pmol/L; 285 pmol/L), respectively). However, mean MMP3, HE4, and CA125 protein levels were significantly higher in the group of patients with ovarian cancer compared to patients with benign ovarian cysts. Statistically significant differences were revealed between mean concentrations of CA125 marker in the group of patients with endometrial cysts (45.2 U/ml) and moderate CA125 levels among patients with benign cysts (19.3 U/ml); *p* = 0.002. With regard to mean TIMP3 concentrations we showed significantly lower values in the group of patients with endometrial cysts compared to patients with benign cysts, *p* = 0.01.

### Analysis of ROC curves, assay sensitivity and specificity

**Fig. 1:**
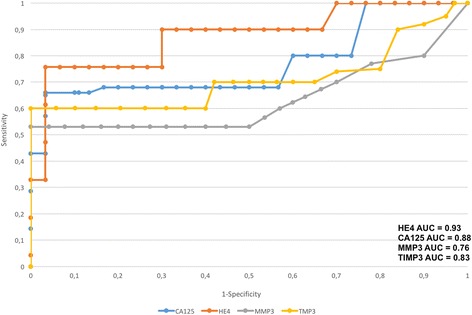
The ROC curve for MMP3, TIMP3, HE4 and CA125 protein in diagnostic patients with ovarian cancer and with benign ovarian cysts without hormonal status division

**Fig. 2:**
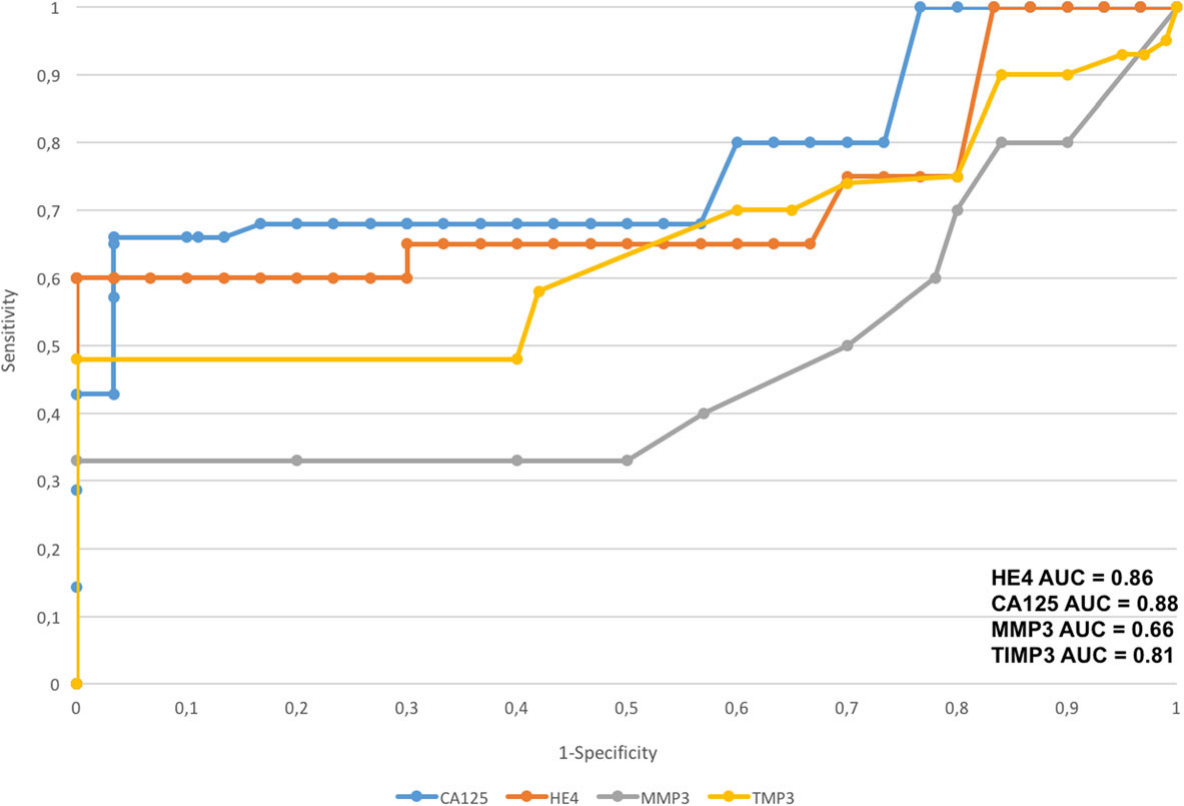
The ROC curve for MMP3, TIMP3, HE4 and CA125 protein in diagnostic patients with ovarian cancer and with benign ovarian cysts for postmenopausal women

In order to evaluate the diagnostic values of MMP3, TIMP3, HE4 and CA125, ROC curves were plotted and the areas under the ROC curves (AUC) were calculated. For HE4 and CA125 the AUC values were 0.93/0.88 for all study patients, 0.92/0.82 for premenopausal patients and 0.86/0.88 for postmenopausal patients. The AUC values for MMP3 and TIMP3 were 0.76/0.83, 0.77/0.82, and 0.66/0.81, respectively (Figs. 1 and 2).

Table 2 presents the sensitivity and specificity values for MMP3, TIMP3 and HE4 and CA125 according to the hormonal status of patients. Higher sensitivity was observed for HE4 in all patients included in the analysis (84%) as well as in postmenopausal patients (86%) compared to TIMP3 (79% and 75%, respectively) and to MMP3 (68% and 66%, respectively) In premenopausal patients, the sensitivity was higher for HE4 (88%) than for TIMP3 (80%), MMP3 (67%) and CA125 (78%). HE4 marker was significantly more specific than MMP3, TIMP3 and CA125 in every study group and amounted to: 92% vs. 69%,78% and 80% in all patients included in the analysis, 94% vs. 70%, 76% and 76% in premenopausal patients, and 85% vs. 65%, 67% and 81% in postmenopausal patients.

**Table 2:** Individual proteins sensitivity and specificity depending on hormonal status

	MMP3	TIMP3	HE4	CA125
sensitivity	68%	79%	84%	75%
specificity	69%	78%	92%	80%
sensitivity PM	67%	80%	88%	78%
specificity PM	70%	76%	94%	76%
sensitivity M	66%	75%	86%	79%
specificity M	65%	67%	85%	81%

### Proteins concentrations analysis according to FIGO clinical progression and histopathological differentiation

Significantly higher mean levels of MMP3 (23,698 pg/mL; 16,542 pg/mL), HE4 (407 pmol/L; 211 pmol/L) and CA125 (2789 U/ml; 1764 U/ml) were also noted among patients with higher clinical staging compared with patients with lower disease staging. Median TIMP3 concentrations were significantly lower among patients with FIGO III and IV staging (109 pg/ml) compared to patients from FIGO I and II groups (198 pg/ml). Statistically significant difference was demonstrated between median MMP3 and HE4 levels among patients with low-grade ovarian cancer compared to well-differentiated ovarian cancer (G1 vs. G3) (*p* = 0.04 and *p* = 0.006, respectively) - Table 3.

**Table 3:** Cox regression analyses of progression free survival and overall survival of classic prognostic factor, MMP3 and TIMP3

	Univariate analysis (Cox regression model)					
	PFS			OS		
	HR	95% CI	*p*-value	HR	95% CI	*p*-value
Age	1.01	0.78–1.08	0.061	1.091	1.01–1.09	0.002
Stage I, II vs. III, IV	3.89	1.73–8.21	0.0001	4.44	1.36–7.98	0.0004
Grade 1 vs. 3	1.09	0.72–2.61	0.1	1.18	0.55–2.26	0.073
Optimal debulking vs. suboptimal debulking	2.07	1.12–4.41	0.0141	1.24	0.57–2.9	0.068
MMP3 - median	1.22	1.01–3.21	0.00023	1.48	0.95–3.73	0.0064
TIMP3 - median	1.68	0.99–4.21	0.00037	1.89	1.21–4.22	0.0073
	Multivariate analysis (Cox regression model)					
	PFS			OS		
	HR	95% CI	*p*-value	HR	95% CI	*p*-value
MMP3 – median	1.14	0.39–1.67	0.0017	1.04	0.78–1,61	0.0001
MMP3–75 percentile	1.03	0.54–2.31	0.08	0.84	0.29–2.76	0.0548
MMP3–95 percentile	1.19	0.15–0.81	0.42	1.04	0.2–0.76	0.007
TIMP3 - median	1.49	0.23–2.68	0.032	1.6	0.43–1.98	0.003
TIMP3–75 percentile	1.01	0.63–3.02	0.005	1.12	0.82–2.21	0.005
TIMP3–95 percentile	1.33	0.88–3.56	0.004	1.092	0.76–3.11	0.03

### MMP3 and TIMP3 concentrations assessment of patients in the individual subgroups

Significantly higher median concentration MMP3 were observed in patients with neoadjuvant chemotherapy as compared to patients in whom primary surgery was possible (*p* = 0.005). On the other hand we have shown statistically significantly lower level of TIMP3 in patients qualified to neoadjuvant chemotherapy (*p* = 0.02) - Table 3. TIMP3 median levels were negatively correlated with ascites in ovarian cancer patients qualified for neoadjuvant chemotherapy (*r* = 0.682, p = 0.02) and for primary surgery (*r* = 0.711. *p* = 0.04). For MMP3 positive correlation was not so strong in the group of patients qualified for surgery (*r* = 0.52, *p* = 0.03).

### Patients survival time evaluation using the Kaplan-Meier curves and Cox proportional hazard regression

Mean baseline TIMP3 levels correlated significantly with total survival time. Patients presenting with values above the median were characterized by total survival time longer by 8.9 months. Mean baseline TIMP3 levels also correlated significantly with disease-free time; *p* = 0.007.

Results were confirmed with Cox regression analysis. For a single grouping variable, we determined a correlation between total survival time of patients and baseline TIMP3 levels (*p* = 0.0073), as well as mean baseline TIMP3 concentrations and time free from the disease (*p* = 0.00037).

By drawing Kaplan-Meier curves we determined that high baseline MMP3 concentrations correlated with total survival time shortened by 9.7 months. Moreover, it was demonstrated that high baseline MMP3 concentrations were significantly associated with shorter disease-free time (*p* = 0.0059). Results were confirmed with the Cox regression analysis of proportional hazards - Table 4.

**Table 4:** Individual proteins concentration levels in patients with ovarian cancer, depending on the staging and grading

		Ascites/ No Ascites	Optimal debulking/neoadjuvant chemotherapy	Low grade/high grade	FIGO I, II/ FIGO III, IV
MMP3 [pgl/ml]	mean	23345/18998	16034/25620	17423/20028	23698/16542
	median	24121/29435	17265/28672	17689/21106	24340/17863
	*p*	NS	0.005	NS	0.004
TIMP3 [pg/ml]	mean	131/203	126/198	204/159	185/122
	median	139/212	129/202	220/161	198/109
	*p*	0.001	0.002	0.03	0
HE4 [pmol/l]	mean	765/542.2	568.3/683.4	180.4/560.3	407/211
	median	771/551	591.4/712.4	198.2/436.3	418/243
	*p*	NS	NS	0.001	0.002
CA125 [U/ml]	mean	1987.2/923.8	672.2/1231	569.2/608.8	2789/1786
	median	2012.4/986.5	689.1/1308	583/703.2	2853/1911
	*p*	0.0003	0.01	NS	0.0037

**Fig. 3:**
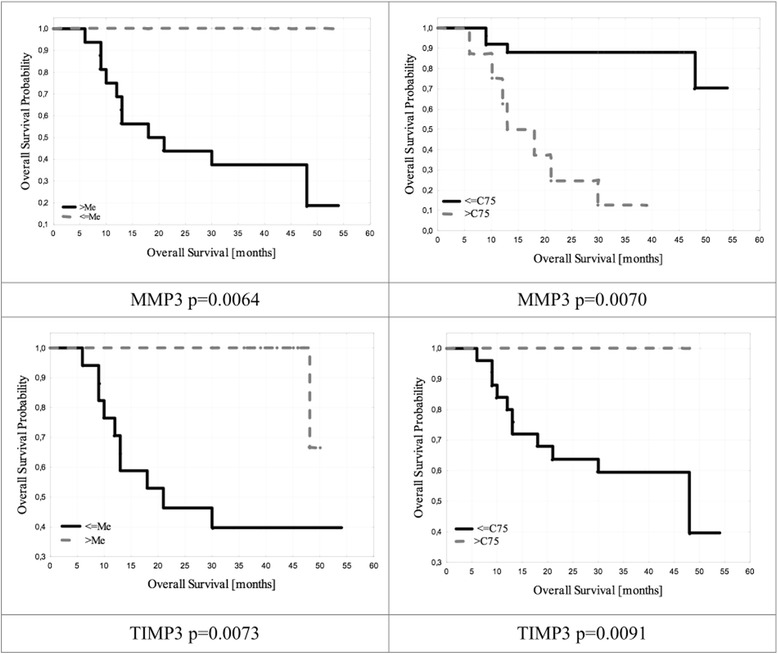
Overall survival stratified by MMP3 and TIMP3 in examined patient with ovarian cancer

Assuming a single grouping variable, we identified a correlation between shorter progression-free time and high baseline MMP3 levels (*p* = 0.00023), as well as between high baseline MMP3 level and total patient survival time (*p* = 0.0064) - Fig. 3.

In the group of patients subjected to neoadjuvant chemotherapy Kaplan-Meier curves demonstrated a statistically significant correlation. Patients with MMP3 levels higher than the median were characterized by total survival time shorter by 11.3 months. In the group of ovarian cancer patients subjected to neoadjuvant chemotherapy Kaplan-Meier curves demonstrated that high baseline TIMP3 levels were associated with total patient survival time longer by 12.4 months.

Taking into consideration the results of HE4 marker assessment after the end of chemotherapy (6 cycles) we demonstrated a statistically significant association in case of patients reaching HE4 values below the median at the end of chemotherapy and total survival time prolonged by 9.6 months.

A statistically significant correlation between level of CA125 marker after the end of chemotherapy and remission/patient survival was shown. Patients with CA125 values above the median were characterized by shorter total survival time; *p* = 0.006. It was also demonstrated that patients with levels of CA125 marker above the median at the time chemotherapy is finished were characterized by disease-free time shorter by 7.9 months.

## Discussion

Extracellular matrix metalloproteinases (MMP) is a family of more than 20 zinc-dependent enzymes. Their primary function is to degrade the extracellular matrix and the basal membrane components. MMPs occur not only in the early stages of tumor development, but in later stages play an important role in tumor invasion and metastasis. Until now, well known MMPs which levels correlate with ovarian cancer are levels of MMP2, MMP7 and MMP9. So far, there is no available information regarding serum level of MMP3 in patients with ovarian cancer and its potential prognostic role. In our study we showed that high baseline mean MMP3 levels were associated with shortened total survival time among patients with ovarian cancer (*p* = 0.00006). Baruch et al. showed an association of matrix metalloproteinase 3 gene expression with the expression level of RAS proteins in cells and tumor’s ability to progress [14]. On the other hand, Choi et al. demonstrated increased expression of MMP3 mRNA in an early stage of ovarian cancer in chickens [15]. In 2004, Denys examined over 12 factors activating or inhibiting tumor invasiveness in colon cancer cell lines and found a striking MMP3 overexpression in tumor cell lines compared to normal fibroblast lines obtained from the same patients. A 50% reduction in invasiveness was demonstrated following administration of a synthetic and physiological MMP3 inhibitor to the cell lines. These results suggested participation of soluble factors released by desmoids cells in stimulation and inhibition of invasion, as well as the role of MMP and TIMP as its mediators Studies by Denys and Zhang demonstrate the role of MMP3 as a factor facilitating invasion by extracellular matrix cells [5, 16]. In such neoplasms as ovarian cancer, which spread by continuity, the role of metalloproteinases seeps particularly important. As we emphasized at the beginning of our discussion, the prognostic role of MMP2 and MMP9 was already investigated.

Both Ozalp and Hu showed that high expression of MMP9 in the extracellular matrix correlated (log-rank test 4.46; *p* = 0.03) with poorer total survival [17, 18]. However, Manenti reported completely different results, showing complete lack of correlation between MMP9 and MMP2 expression in the tissues and patient total survival or disease-free time [19]. Desmeules et al. found that elevated tissue expression of MMP9 is related to total survival time in women, while MMP2 overexpression has no association with disease-free time and survival among patients with ovarian cancer [20].

Matrix metalloproteinase inhibitors are supposed to directly regulate angiogenesis by acting on MMP, but also through direct inhibition of angiogenesis activators.

TIMP3 exerts an anti-angiogenesis effect by inhibiting neoplastic proliferation and migration through MMP protein inhibition. It was proven that TIMP3 overexpression led to apoptosis in vitro and increased apoptosis in human breast cancer cells (MDA-MB435) [21].

In our study we determined the concentrations of metalloproteinase 3 inhibitor (TIMP3) in the context of possible use of changing concentrations of the factor in predicting disease course. We demonstrated a statistically significant correlation between TIMP3 protein levels and patient total survival time. Moreover, it was shown that high median TIMP3 levels at baseline were significantly correlated with longer disease-free time (*p* = 0.00773). However, Hu et al. found elevated TIMP3 expression among patients with ovarian cancer and lack of correlation between expression of TIMP3 mRNA and total patient survival time [18]. In 2016 Das et al. demonstrated that TIMP is a dominating negative regulator of angiogenesis in malignant melanoma; low levels of this factor are associated with poor prognosis. There was significant inverse correlation between TIMP3 expression and blood vessel density in the tumor (*p* = 0.031) [22].

Some authors emphasize a unique role of TIMP3 as a tumor suppressor. TIMP3 exhibits the ability to inhibit tumor angiogenesis, invasion, and metastasis. In a study by Gu et al. correlation between TIMP3 expression and disease progression in HCC tumors was demonstrated, including portal vein infiltration (*p* = 0.036) and lymph node metastases (*p* = 0.030). Cox regression analysis conducted on the study group revealed that TIMP3 expression was an independent prognostic factor for disease-free survival (*p* = 0.039) and total survival (*p* = 0.049) [23]. Manenti et al. reported different results, showing a relationship between TIMP1 expression and poorer prognosis in ovarian cancer patients. On the other hand, Brun et al. in their studies on MMP2, 7 and 9 expressions as well as the expressions of tissue inhibitors, such as TIMP1 and 2, used Kaplan-Meier analysis to demonstrate lack of correlation between the expression of those proteins and survival in a group of patients with ovarian cancer [24].

Moreover, in our study we strive to answer a common question regarding CA125 protein. When should the levels of this protein be determined in order to constitute the best possible prognostic factor in ovarian cancer patients? In our research we demonstrated that mean concentrations of CA125 marker at the end of chemotherapy are associated with total survival (*p* = 0.002) and disease-free time (*p* = 0.03).

Likewise, Kim et al. used Kaplan-Meier curves and Cox regression analysis to demonstrate that serum CA125 levels after 6 cycles of chemotherapy might constitute a prognostic factor for total survival among patients with advanced ovarian cancer. Other studies by the same research team showed that serum CA125 level after the end of chemotherapy was a strong prognostic factor in a group of high-risk patients with low-grade ovarian cancer [9]. Concentration of CA125 marker <10 U/ml after the end of chemotherapy is considered an independent prognostic factor regardless of the size of postoperative remnants [25].

Moreover, we determined the levels of HE4 protein. Results we obtained are concordant with those acquired by Piovano. In a review concerning the role of HE4 as a predictive and prognostic factor Piovano emphasizes that lack of HE4 normalization at the end of standard therapy might indicate poor prognosis and suggesting the need for closer follow-up in this group of patients. These reports are in line with our results, demonstrating that higher HE4 levels after the end of treatment correlated with shorter progression-free time [11].

Our research shows that neither metalloproteinase 3 nor its inhibitor should be routinely used in the diagnosis of ovarian cancer, as they do not exceed the specificity or sensitivity of the markers currently used. However, we believe that introducing additional markers for serum level of MMP3 and TIMP3 as prognostic factors could help to individualize treatment of the patients.

## Conclusion

Preliminary studies indicate that baseline MMP3 and TIMP3 concentrations are associated with patient survival and disease-free time. It constitutes the basis for further studies on those proteins in the context of their prognostic significance. Both of these proteins do not satisfactorily meet the criteria of good diagnostic testing, regardless of the hormonal status of patients.

## Abbreviations

AUC: Area under the curve
CA125: Carbohydrate antigen 125 (cancer antigen 125)
FIGO: Fédération Internationale de Gynécologie Obstétrique (International Federation of Gynecology and Obstetrics)
G[1–3]: Grading
H: High grade
HE4: Human epididymis protein 4
L: Low grade
M: Menopausal status
MMP3: Matrix metalloproteinase-3
OS: Overall survival
PFS: Progression free survival
PM: Premenopausal status
ROC: Receiver operating characteristic curves
TIMP3: Tissue inhibitor of metalloproteinase 3
